# Innovative Fatty Acid-Guided Biosensor Design for Neutrophil Gelatinase, a Prognostic and Diagnostic Biomarker for Chronic Kidney Disease

**DOI:** 10.3390/bios16020074

**Published:** 2026-01-26

**Authors:** Kaustubh Jumle, Priya Paliwal, Mohamed A. M. Ali, Ravi Ranjan Kumar Niraj, Anis Ahmad Chaudhary, Manali Datta

**Affiliations:** 1 Amity Institute of Biotechnology, Amity University Rajasthan, Jaipur 303002, India; 2 Department of Biology, College of Science, Imam Mohammad Ibn Saud Islamic University (IMSIU), Riyadh 11623, Saudi Arabia

**Keywords:** chronic kidney disease, neutrophil gelatinase, linoleic acid, electrochemical detection, specificity

## Abstract

Chronic kidney disease (CKD) afflicts 850 million people worldwide, with an estimate that it is the 5th highest cause of years of life lost (YLLs). Standard confirmatory procedures for disease are blood and urine analysis with ultrasound for confirmation. Neutrophil gelatinase-associated lipocalin (NGAL) has been established as a prognostic biomarker, especially for the pre-clinical stages of CKD, thus presenting itself as a dependable predictor of the progression. With the aim of designing diagnostics, fatty acids were explored as potential biorecognition elements for the selective capture of NGAL. Three fatty acids—linoleic acid, arachidonic acid, and retinoic acid—were shortlisted as plausible candidates based on their known affinity toward lipocalin family proteins. Docking followed by molecular dynamics simulations were employed to evaluate the binding affinity and stability of each complex. Among them, linoleic acid exhibited the most favorable interaction, as evidenced by the lowest binding free energy. Subsequently, fluorescence and electrochemical techniques—square-wave voltammetry, differential pulse voltammetry, cyclic voltammetry, and electrochemical impedance spectroscopy (EIS)—were systematically compared for qualitative and quantitative checking of the accuracy of NGAL detection. Amongst the electrochemical techniques, differential pulse voltammetry DPV demonstrated superior analytical performance with an LOD of 0.05 ng/mL with a sensitivity of 23.2 µA/cm^2^/pg. To the best of our knowledge, this is the first report of a fatty acid-based biosensor platform for NGAL detection, presenting a novel approach for CKD diagnostics. The sensitivity obtained is comparable with available NGAL detection methods yet cost-effective and robust.

## 1. Introduction

Chronic kidney disease (CKD) presents as a potent cause of anemia and cardiovascular complications, with eventual progression to hormonal imbalances and tuberculosis. The symptomatic presentation of CKD is unanticipated and primarily characterized by differential levels of damage to tubular regions of nephrons [1,2]. Diagnosis of CKD is exhaustive, comprehensive, and reliant on clinical examinations involving blood panels, physical tests and imaging assessments. Standard confirmatory procedures for CKD include inulin-based glomerular filtration rate and serum creatinine-, urea-, and ultrasound-based diagnostics for analyzing the renal parenchymal damage [3]. CKD is clinically diagnosed with symptomatic presentations, whereby the progression is characterized by renal function decline (Stage I-II), physiological manifestations (Stage III-V), and end stage renal failure (ESRD). ESRD has two lifesaving choices—dialysis and renal replacement therapy. Based on a microsimulation assessment for the prevalence of CKD, the number is expected to rise to 436.6 million cases by 2027 with a rate of 5.8% unless an early diagnosis is implemented [4]. No single diagnostic can confirm if a person has CKD; hence, there is always an avenue for developing ancillary biomarker-based diagnostics for CKD. A modeling study consisting of four European countries estimated that early detection will assist in saving about EUR 15.8 billion over a 10-year period in terms of medical costs [5,6].

Lipocalins are a class of secreted proteins that are capable of transporting small lipophilic molecules [7] and possess roles in cell homeostasis, pheromone transport, and olfaction [8]. A member of the lipocalin family, Neutrophil gelatinase-associated lipocalin (NGAL), acts as a transporter of prostaglandins and fatty acids (FAs) [8]. It is also an established prognostic and diagnostic biomarker for chronic kidney disease and, more recently, COVID-19-induced kidney malfunction [9,10].

While several diagnostic platforms have been developed with FAs as target analytes [11], there are currently no established diagnostic systems that utilize them as immobilized ligands. Lipocalins possess an inherent affinity for FA owing to its structural configuration [12] and thus FA may be used as a suitable probe for NGAL. This gap presents a novel and underexplored strategy for biosensor design, wherein the specific molecular recognition between FA and target proteins may be exploited to develop next-generation diagnostic platforms with enhanced specificity and stability.

NGAL has been proven to be a binding partner for fatty acids [12]; hence with the aim of designing diagnostics, FAs were considered as plausible bait for the capture of NGAL. Amongst these, linoleic acid, arachidonic acid, and retinoic acid were selected as probable candidates. Although immunosensors for the detection of NGAL have been designed [13], they have distinct disadvantages associated with them like antibody instability and higher cost with limited shelf life and batch variations [14]. This is the first time that an FA-based diagnostic has been proposed for the detection of NGAL which overcomes all the drawbacks associated with immunosensors. This paper, ascertains the best bait for NGAL computationally and, and confirms its further in vitro applicability in diagnostic development via electrochemical analysis.

## 2. Materials and Methods

### 2.1. Materials

Screen-printed carbon electrodes (SPCE–REF: ItalSens IS-C) were purchased from Palmsens, Houten, Netherlands. Linoleic acid (LA), Neutrophil gelatinase-associated lipocalin (NGAL), Sodium hydroxide (NaOH), Poly-L-Lysine, 1-Ethyl-3-(3-dimethyl-aminopropyl) carbodiimide (EDC), N-hydroxysuccinimide (NHS), and 11-(dansylamino) undecanoic acid (DAUDA) were purchased from Sigma Aldrich, St. Louis, MO, USA. 3-Aminopropyl triethoxysilane (APTES) was procured from Alfa Aesar, Ward Hill, MA, USA. Potassium hexacyanoferrate (III) [K_3_Fe(CN_6_)], Ethanol, and other chemicals were obtained from Qualigens, Mumbai, India, and were of analytical grade.

For bioinformatics analysis, coordinate information of NGAL (1DFV) was downloaded from RCSB-PDB (www.pdb.org, accessed on 16 December 2024) [15] in .pdb format. The quality check of the protein structure file was performed in PROCHECK v.3.4.5 (https://saves.mbi.ucla.edu/, accessed on 18 December 2024) [16]. The file was cleaned and processed for further docking using the edit tool of AutoDock 1.5.6 [17]. The structure data files for all fatty acids considered in the study were downloaded from the PubChem database of clinical molecules [18]. The files were downloaded in .sdf format and PyMol 2.5.4 (“PyMOL|Pymol.Org,” n.d.) followed by AutoDock 1.5.6 [17] was used to convert the files into docking essential .pdbqt format.

### 2.2. In Silico Assessment for Identification of Best FA Bait Using Molecular Docking and Molecular Dynamics Simulation

AUTODOCK Vina 1.1.2 [17], a protein–ligand docking tool, along with the Lamarckian Genetic Algorithm was used to dock against the NGAL protein, to minimize processing time while increasing the degree of freedom, resulting in superior outputs even with complicated ligands. For the production of the grid box and subsequent calculations of the energy grid maps, the Auto Grid program in AutoDock package was utilized. The package’s AutoDock program searches for conformations and generates energy estimates. During processing, polar hydrogens were added to the protein with appropriate Kollman charges. The grid box was set to 70.527 × 26.383 × 61.100 (x, y, and z) and the grid point spacing was kept at the default value of 0.375 Å. The top poses were selected based on the affinity energies that were obtained upon successful docking of the fatty acids with NGAL. The complexes were submitted to structure-based analysis using visualization software PyMol (“PyMOL|Pymol.Org,” n.d.). The top conformers were analyzed using PyMol and 2D interaction was generated using Ligplot 2.3 [19]. Ligplot was also utilized to identify interacting amino acids that bind via hydrogen bonds and hydrophobic interactions. The Steepest Descent approach was used to minimize the energy of the complexes using the SwissPDB4.1.0 viewer [20]. A molecular dynamics (MD) simulation was performed using the GROMACS 2018.1 package [21] with forcefield Gromos 43A1. The protein solvation was performed by an SPC water model in a cubic box (10.8 × 10.8 × 10.8 nm^3^). The energy was minimized up to a maximum of 25,000 steps or until the maximum force (Fmax) was less than 1000 kJ/mol/nm (default threshold) by using the steepest algorithm. NVT and NPT with 50,000 steps, 100 ps at 300 K, and 1 atm were performed. The equilibration of the complete system and the final MD simulation was performed for 50 ns with the calculation of RMSD [21,22].

### 2.3. Investigative Fluorescence Study for LA-NGAL Interaction

Preliminary bioinformatics assessments indicated LA to be the best interacting partner with the least potential energy, making it a ligand of high interest. Precision of the MD simulation was tested using a spectrofluorometer (Jasco FP-8250, Easton, MD, USA) at room temperature with an excitation wavelength of 345 nm, and the emission was observed from 350 to 650 nm set for DAUDA [23,24]. DAUDA (3 mg/mL) diluted in PBS was used with different permutations of NGAL and LA to ascertain its competitive binding to NGAL.

### 2.4. Electrochemical Analysis of the LA-NGAL Interaction

A probe-based electrochemical sensor platform was postulated to analyze NGAL in bodily fluids. Preparation of the electrochemical platform was designed as per the protocol followed by Paliwal et al., 2022 [22]. Briefly, a working electrode of screen-printed carbon electrodes (SPCEs) was activated using Poly-L-Lysine, whereby an SPCE was initially rinsed with distilled water and a brief CV cycle for 5 s followed by air drying. A 0.05% Poly-L-Lysine solution was then incubated for 6 hrs followed by water rinsing and air drying. A mixture of EDC-NHS and LA was incubated overnight at RT for immobilization (LA-SPCE). Formation of an LA-SPCE was monitored using scanning electron microscopy (SEM) (Tescan MIRA, Brno, Czech Republic) and Fourier Transform Infrared Spectroscopy (FTIR) (Nicolet iS5, Thermo Fischer Scientific, Ann Arbor, WI, USA). Four electrochemical techniques were assessed to check the sensitivity of the proposed diagnostic platform, namely, square-wave voltammetry (SWV), cyclic voltammetry (CV), differential pulse voltammetry (DPV), and electrochemical impedance spectroscopy (EIS).

The LA-SPCE was incubated with 2–10 μg/mL NGAL for an incubation period of 10 min. SWV (Palmsens 4, Utrecht, The Netherlands) was performed using a basal frequency of 1.5 Hz and an amplitude of 0.05 V, with a potential scan in the range of −400 mV to 400 mV with a step increment of 10 mV. A 10 s equilibration was employed before each measurement. CV assessed the oxido-reduction potential of LA-NGAL with increasing biomarker concentration with an incubation time of 15 min, using 5 mM potassium ferricyanide [K_3_Fe(CN_6_)] as an indicator. The experimental parameters were set with potential sweeping between −600 mV and 600 mV at a scan rate of 70 mV/s and E_step_ of 10 mV. Similarly, parameters for DPV were fixed with start and end potentials as −400 mV to 400 mV at a scan rate of 100 mV/s with an E_step_ of 10 mV and E_pulse_ of 20 mv in the range of 1–10 ng/mL. The range for EIS was set between 100 Hz and 100 kHz with a perturbation amplitude of 10 mV and DC bias potential (E_dc_) of 0.20 V. The sensitivity and LOD were calculated via the calibration curve plotted between the charge transfer resistance (R_ct_) versus varying concentrations of NGAL.

### 2.5. Cross-Reactivity Analysis

As the objective of this study was to develop a sensor platform amenable to translation into real-world clinical settings, evaluation of cross-reactivity of the designed platform was essential. Accordingly, to mimic the probable proteins and biomarkers [24] present under CKD conditions, the LA–SPCE was tested against potential interferents like α2-macroglobulin, urea, and creatinine, each at a concentration of 5 ng mL^−1^, under identical experimental conditions. Signal generation was quantified relative to the LA–SPCE baseline response, which was used as the control.

### 2.6. In Vitro Validation of the Developed Platform

Efficacy of the fabricated platform was tested in spiked urine samples. Urine was collected from a healthy volunteer, filtered to remove suspended particles and was spiked ex vivo with known concentrations of the target marker. The spiked urine was then tested on the pre-processed platform to assess sensor performance under realistic conditions. Post 15 min of incubation, DPV measurements were performed and the specificity and sensitivity of the platform was calculated using the following formula [25]:

Sensitivity = Slope/Working Area of electrode(1)

LOD = (3 × standard deviation)/sensitivity(2)

## 3. Results

### 3.1. Molecular Docking and Dynamics to Assess the Binding of NGAL and FAs

The human NGAL protein 3D structure (1DFV) was obtained from RCSB-PDB; overall acceptability of the protein model was assessed by PROCHECK, where the Ramachandran plot designated almost 97.4% of amino acids falling within the most favorable regions. The initial forcefield energy was calculated to be −5091.695 KJ/mol using SPDBV v4.0. Following energy minimization, reduction in inherent energy was obtained (−8043.835 KJ/mol) and this energy stabilized the 3D structure was used for docking. Template-free docking was performed for NGAL with RA, LA, LnA, MA, and AA; affinities based on their binding energies were computed. RA had the highest affinity with an energy of −6.4 kcal/mol, followed by LA and AA, both at −5.3 kcal/mol (Table 1).

**Figure 1:**
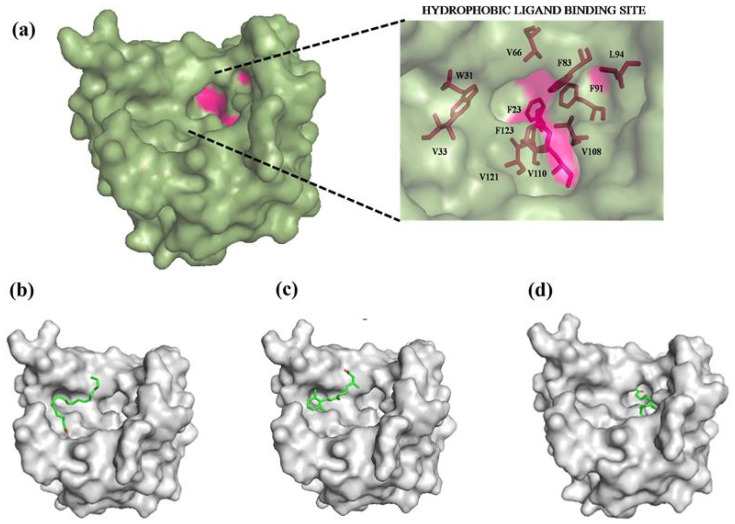
(**a**) Lipophilic ligand binding pocket highlighted in pink with the amino acids constituting the substrate binding site. Docked structures of (**b**) arachidonic acid, (**c**) retinoic acid, and (**d**) linoleic acid with NGAL. All lipocalins have lipocalin fold, beta barrel and hydrophobic pocket [26] W31, T113, and R140, along with the 310 helix and a hydrophobic pocket encompassing F27 from 310 helix, W31 and V33 from β1, V66 from β3, F83 from β4, F92 and L94 from β5, V108 andV110 from β6, V121 and F123 from β7. In contrast, positively charged amino acids like R81, K125, and K134 facilitate binding of hydrophilic ligands [27]. Furthermore, the 3D complex structure of LA-NGAL revealed LA to be stabilized with hydrophobic interactions with Gly38, Ile41,Val66, Leu70, Trp79, Phe83, Phe123, and Phe133 residues which coincide with the lipophilic substrate binding site present in NGAL. Furthermore, Arg81, Lys125, and Lys134 indicated a stabilization effect on LA in the shallow end of the NGAL calyx.

Analysis of the docked structure of NGAL binding of the different fatty acids, LA presented itself as the best-fitting ligand (Figure 1b–d). A reduced ligand strain was evident with the energy value corresponding to −8994.246 KJ/mol for the LA-NGAL complex as compared to −8843.835 KJ/mol for an energy-minimized structure of NGAL [25].

Results from backbone RMSD (Figure S1) plotted as a function of time indicated the complex was stable after commencing the simulation. The RMSD values of NGAL and NGAL-LA seemed to vary [27,28] after 40 ns, with the RMSD trajectory varying from 12 Å to 35 Å. Based on the dry lab analysis, LA was selected as the probe for generating the sensor platform.

### 3.2. Interaction Study for LA-NGAL Using Fluorescence

**Figure 2:**
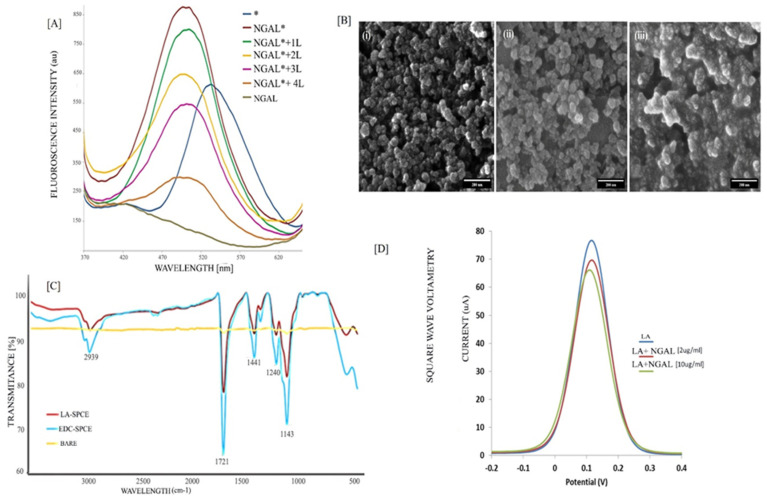
Biophysical characterization for LA-NGAL and LA-SPCE. (**A**) Fluorescence spectra showcasing the competitive binding study of fatty acid and protein using DAUDA [*] as a competitive indicator. (**B**) SEM micrograph of (**i**) SPCE, (**ii**) LA-SPCE, and (**iii**) LA-NGAL SPCE (magnification: 200,000×). The bare SPCE electrode exhibited a fairly porous structure, while the LA-SPCE electrode showed a decrease in apparent pore size and surface roughness, consistent with effective LA immobilization. (**C**) shows the comparison of FTIR spectra with prominent intensity, indicating the presence of -COOH, -NH_2_, and -C-C- groups (yellow—bare SPCE; blue—EDC-SPCE; red—LA SPCE). (**D**) SWV sweep electrochemical testing of LA-SPCE for biomarker NGAL (blue—Ip for LA-SPCE; maroon—spiked buffer containing 10 ng NGAL; green—spiked buffer containing 10 ng NGAL).

DAUDA binding to NGAL was associated with a considerable rise in fluorescence intensity and a shift in the maximum fluorescence emission wavelength from 535 nm to 500 nm, indicating entry into a strongly apolar environment. Upon addition of LA in the mixture of protein and DAUDA, there was a decrease in the observed fluorescent intensity. The intensity of the peak decreased as the LA concentration rose (Figure 2A), indicating a clear demarcation in fluorescence.

### 3.3. Biophysical Characterization of Working Electrode

Surface activation of the WE was analyzed using FE-SEM (Figure 2B) and FTIR (Figure 2C). FE-SEM imaging was carried out at an accelerating voltage of 10 keV using the InBeam secondary electron (SE) detector (Tescan MIRA, Brno, Czech Republic) The working distance was maintained between 4.74 and 4.91 mm, and images were acquired at a magnification of 200,000×. The obtained micrographs were subsequently processed and analyzed using ImageJ software 1.54. The surface morphologies of the SPCE, LA-SPCE, and LA-SPCE-NGAL were analyzed using FE-SEM; the SPCE (Figure 2B(i)) electrode exhibited a fairly porous structure, while the LA-SPCE (Figure 2B(ii)) showed a significantly reduced pore size, indicating successful surface modification and partial pore blockage due to the immobilization process. The NGAL-LA (Figure 2B(iii)) was associated with further smoothing and densification of the surface with clustered features attributed to protein adsorption.

An FTIR spectrum corresponding to the bare electrode indicated a constant line with no peaks (Figure 2C). The coating of LA was perceptible by the presence of a peak at 2939 cm^−1^ corresponding to the -OH stretching (carboxyl groups) and -C-H stretching vibrations that are associated with LA. Significant peaks at 1721 cm^−1^ and 1143 cm^−1^ correspond to C=O stretching vibrations of COOH and C-NH_2_ primary amine, respectively [Figure 2C]. Amide bond formation between -NH_2_ and -COOH of LA was confirmed by the presence of a peak at 1441 cm^−1^. Other peaks corresponding to -CH_2_ stretching were observed at 2939 cm^−1^ and C-C stretching and -OH bending corresponding to FA were observed at 1441 cm^−1^, 1240 cm^−1^, and 1384 cm^−1^ [25].

### 3.4. Electrochemical Analysis of the Working Platform

The effect of LA-NGAL interaction was measured using 5 mM K_3_FeCN_6_ as a redox indicator; working concentrations of NGAL in the range of 5 ng/mL to 50 ng/mL were used for all the electrochemical analyses in the three electrode systems (ItalSens Carbon SPE).

SWV studies for bare LA-SPCE shows a high peak current (Ip) in comparison to the lipocalin exposed electrode (Figure 2D); Ip significantly decreased with increase in NGAL concentrations. An amount of 10 ng of protein resulted in a 7 µA decline with an additional dip of 3.4 µA with 50 ng of NGAL. As a result, the overall decline in current from the highest value of 76 µA was determined to be 10.32 µA.

**Figure 3:**
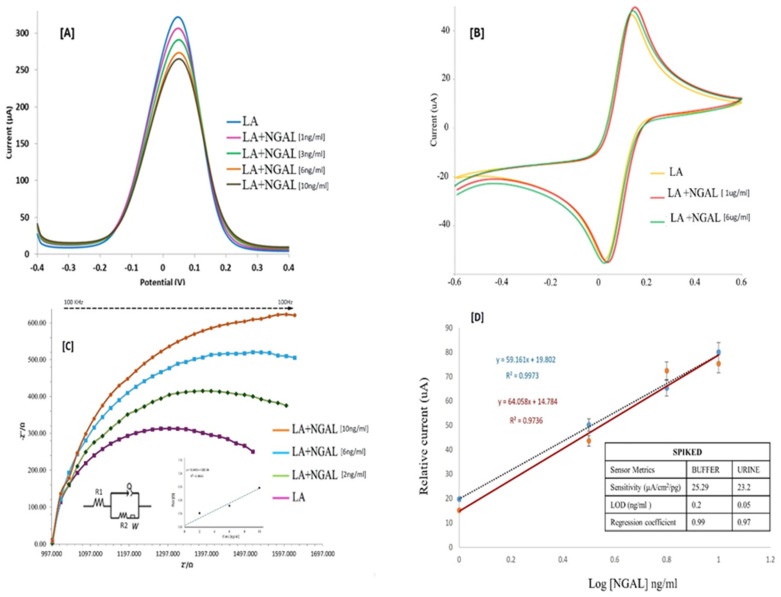
(**A**) DPV, (**B**) CV, and (**C**) EIS curves of LA-SPCE AND LA-NGAL interactions. (**C**) Inset represents the Randles circuit and the calibration curve for EIS. The electrolyte used was K_3_[Fe(CN)_6_] (5 mmol L^−1^) using spiked buffer sample. (**D**) Calibration curve for LOD calculation using DPV for the spiked urine sample in the range of 5–50 ng NGAL, whereby the red solid line represents the spiked urine sample and the blue dotted line is the spiked buffer with marked data points.

In DPV, the highest current output was obtained at 322 µA (Figure 3A) for the LA-SPCE. With 10 ng/mL NGAL, the current output was obtained around 265 µA, indicating a sharp decrease of approx. 57 µA in total. The calibration curve represented in Figure 3D gives us the equation of y = 58.8x + 19.9 where y is the current output associated with the biosensor and x is the concentration of NGAL. The R^2^ value concomitant with the linear fitting is 0.99, indicating that best fit passes near to our data points. The overall LOD for this biosensor was calculated to be 0.05 ng/mL with a sensitivity of 23.2 µA/cm^2^/pg. Additionally, operational stability of the sensor was assessed by monitoring DPV results for NGAL (5 ng mL^−1^) on different electrodes and after storage at 4 °C for 15 days. The sensor retained more than 90% of its initial signal after 15 days, indicating good stability of the sensing interface (Figure S2). The DPV readouts obtained from different electrodes gave a maximum variability of 3 µA.

The CV profile (Figure 3B) shown for the NGALincubated LA-SPCE indicates an oxidative peak, whereby LA induces a forward electron transfer to NGAL resulting in peak currents of +49.440 µA and −58 μA at +0.18 V and −0.020 V, respectively. However, the differences between the peaks at varying concentrations are not perceptible enough to be reliably distinguished, which may limit CV for identifying stages in a point-of-care device.

Although both LA and NGAL are intrinsically insulating, formation of the LA–NGAL complex induces reorganization of interfacial charge distribution at the electrode surface. These electrostatic rearrangements enhance double-layer charging and capacitive contributions that are captured in CV, which integrates both faradaic and non-faradaic currents. Importantly, this response is not associated with enhanced electron transfer through the bio-layer, as confirmed by the absence of corresponding signal enhancement in DPV [28]. Thus, the CV response reflects interfacial electrostatic reorganization rather than improved conductivity.

EIS measurements were performed in the frequency range of 100 Hz–100 kHz, with a voltage amplitude of 0.01 V for LA-NGAL, and plotted on a Nyquist plot and fitted in a Randles equivalent electrical circuit model (Figure 3C). For a Randles circuit, each component corresponds to a specific aspect of the physical structure at the electrode/analyte interface. The semicircular region observed in the Nyquist plots, indicative of electron transfer kinetics, was modeled using a parallel configuration of a resistor (R_2_) and a constant phase element (CPE). Diffusion-limited electrochemical processes were modeled by the Warburg element (W), whereas the solution resistance was represented by R_1_. EIS measurement indicated a concomitant increase in the charge transfer resistance due to steric hindrance that was brought about by the bulky protein molecule. The analytical method was validated by the determination of the LOD. A good linearity was obtained with an LOD of 13.6 ng/mL.

For our sensor platform, it was observed that DPV is more sensitive than EIS. DPV measures faradaic peak current, sensitive to minor perturbations in electron transfer kinetics, meaning it could detect low concentrations of NGAL, whereas EIS measures changes in Rct arising from progressive surface blocking after NGAL binding to LA. Consequently, DPV exhibits enhanced sensitivity at lower analyte concentrations, whereas EIS demonstrates linearity at comparatively higher NGAL levels despite identical incubation conditions.

### 3.5. Cross-Reactivity and in Vitro Validation of LA-SPCE for NGAL Detection

Electrochemical measurements using DPV demonstrated negligible cross-reactivity (Figure S3), with non-target interferents producing insignificant signal changes relative to the NGAL-specific response. DPV measurements using spiked urine samples gave good relative readings for higher concentrations, thus indicating competent performance for in vitro measurements (Figure 3D). The calibration curve for the spiked buffer demonstrated higher sensitivity and lower background contribution. The absence of competing species in buffer minimally hindered electron transfer. In contrast, for urine, an altered slope and intercept indicate a matrix effect, due to salts and other metabolites influencing charge transfer and surface interactions. Despite this, the linear response of the calibration curves for spiked buffer and urine demonstrates that the LA-SPCE retains its analytical performance in a real biological matrix with minimal signal distortion. The LOD for the LA-SPCE with spiked urine was calculated to be 0.2 ng/mL with a sensitivity of 25.29 µA/cm^2^/pg. The higher LOD in urine arises due to unavoidable matrix interference, which is common for real biological samples (Figure 3D inset). This data along with cross-reactivity results indicate that the LA-SPCE may be translated for commercial usage.

## 4. Conclusions

To date, no point-of-care diagnostic has been proposed which might detect CKD in home settings. The clinical significance of NGAL arises from its intense accumulation in approximately 50% of cortical tubules in patients at risk of developing or already experiencing moderate to severe kidney injury. This pronounced immunoreactivity underscores NGAL’s utility as a sensitive and prognostic biomarker of renal tubular damage [23,25], a precursor for CKD.

**Scheme 1:**
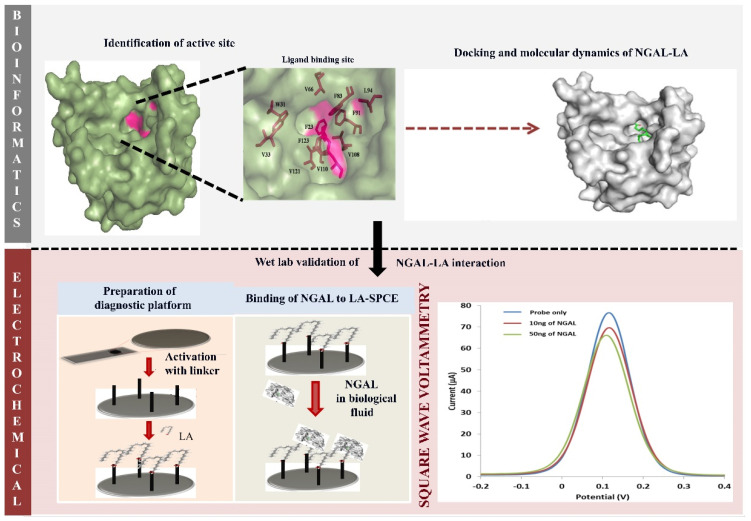
Integrated bioinformatics and electrochemical validation of NGAL–linoleic acid (LnA) interaction. A bioinformatics workflow was used to identify the ligand-binding site of NGAL, whereby key amino acid residues involved in the stable binding of LA within the NGAL cavity were established. Using LA as an immobilized probe, electrochemical validation of the NGAL–LnA interaction was assessed. Concentration-dependent changes in current response were observed upon exposure to increasing NGAL levels, confirming successful recognition and binding on the sensor surface.

A diagnostic design was conceptualized (Scheme 1) for the detection of NGAL as a biomarker for screening of CKD candidates. In silico and in vitro experimentation confirmed that FAs serve as good candidates for binding to NGAL; as the intended diagnostic prototype is conceptualized based on electrochemical interaction, NGAL-LA presented as a more reliable target–probe combination. As NGAL concentration increases with progressive CKD, the platform was evaluated for fluctuations with increasing concentrations of NGAL. The use of LA enabled competitive analytical performance, with our platform achieving an LOD of 0.05 ng/mL using DPV and 13.6 ng/mL via EIS. Thus, the diagnostic platform designed on the complementary interaction using NGAL as a biomarker and LA as an optimized ligand may supplement prognostic detection of CKD and add to the armamentarium of CKD in vitro diagnostics.

NGAL biosensors (Table 2) have previously been developed [29,30,31], primarily utilizing antibodies as ligands, achieving LODs in the range of 0.6 pg/mL–4.59 ng/mL. However, the sensitivity and robustness of such platforms are highly dependent on the antibody’s affinity and stability. In contrast, our biosensor platform presents a distinct advantage through the selection of LA as the recognition element, with a comparable LOD. Unlike monoclonal antibodies, which can cost between USD 50–100 per gram, the use of LA significantly enhances the cost-effectiveness of the sensor without compromising performance, thereby paving the way for the development of next-generation biosensors guided by specific molecular recognition mechanisms.

## 5. Patents

Provisional Patent in the Indian Patent Office: A Poly Unsaturated Fatty Acid Based Sensor Platform System To Capture Disease Specific Biomarker: 202211002786 [E1/2938/2022-DEL].

## Figures and Tables

**Table 1:** Docking Energy for Different Fatty Acids.

Fatty Acid	Docking Energy (cal/moL)
Arachidonic acid	−5300
Linoleic acid	−5300
Retinoic acid	−6400

**Table 2:** Comparative for NGAL Sensors.

SENSOR	TRANSDUCTION	LOD [ng/mL]
NGAL Aptamer-Based Electrochemical Aptasensor [32]	SWV/EIS	0.48–4.59
Electrochemical Aptasensor (NGAL Aptamer + DNAzyme) [33]	DPV	~4.45
Peptide-Based Electrochemical Sensor [34]	EIS/SWV	~1.74–3.93
Electrochemical Immunosensor [35]	Amperometry	~1
Optical Immunobiosensor [35]	Photoelectrochemical	~0.0006
NGAL FA sensor [Current study]	DPV	0.05

## Data Availability

No new dataset was created.

## Supplementary Materials

The following supporting information can be downloaded at https://www.mdpi.com/article/10.3390/bios16020074/s1, Figure S1: Binding mode and corresponding molecular dynamics simulations of NGAL with LA, where the MD trajectory results depict the root-mean-square deviation (RMSD) in a 50 ns timeframe; Figure S2: Operational stability of the sensor over 15 days. Mean activity (±SD, n = 3) recorded at regular intervals (days 1, 3, 6, 9, 12, and 15). The dashed line indicates the linear trend, showing only a marginal decline in activity over time; Figure S3: Selectivity of LA sensor for biomarkers present in urine. LA–SPCE sensor was tested against potential interferents, including α2-macroglobulin, urea, and creatinine, each at a concentration of 5 pg mL^−1^, under identical experimental conditions.
