# Supplementary material for: Innovative Fatty Acid-Guided Biosensor Design for Neutrophil Gelatinase, a Prognostic and Diagnostic Biomarker for Chronic Kidney Disease
Source: Biosensors (Basel). 2026 Jan 26;16(2):74. doi: 10.3390/bios16020074 (PMC12938534; doi:10.3390/bios16020074)
Supplement: Supplementary file 1 [file biosensors-16-00074-s001.zip › biosensors-4027890-supplementary.pdf]

SUPPLEMENTARY FILE

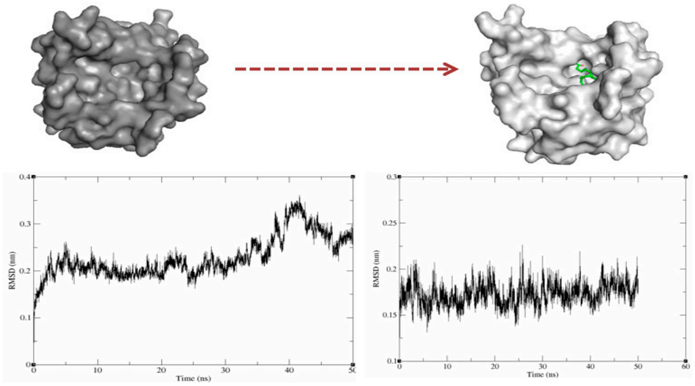

Figure S1: Binding mode and corresponding molecular dynamic simulations of NGAL with LA where the MDs trajectory results depict the root-mean-square deviation (RMSD) in a 50ns timeframe

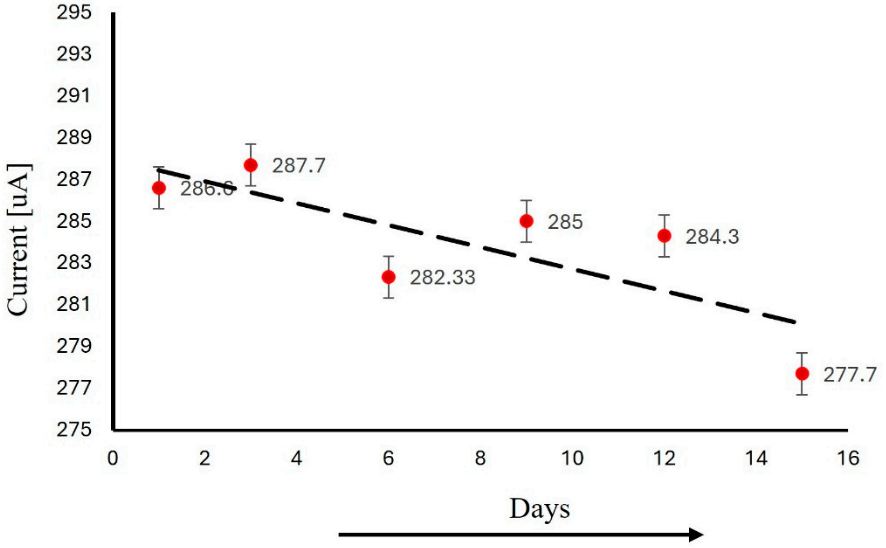

Figure S2: *Operational stability of the sensor over 15 days.* Mean activity (± SD, n = 3) recorded at regular intervals (days 1, 3, 6, 9, 12, and 15). The dashed line indicates the linear trend, showing only a marginal decline in activity over time

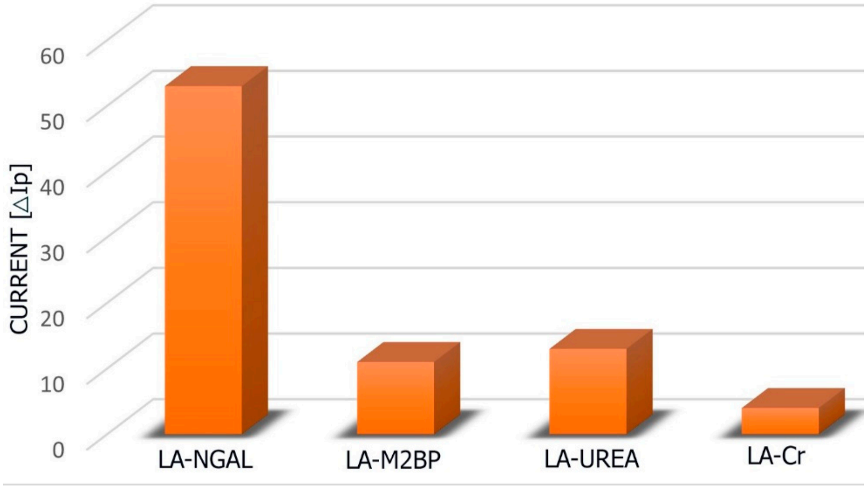

Figure S3: Selectivity of LA sensor for biomarkers present in urine. LA–SPCE sensor was tested against potential interferents, including α2-macroglobulin, urea, and creatinine, each at a concentration of 5 pg mL^{-1}, under identical experimental conditions
